# Correction: Cyborg groups enhance face recognition in crowded environments

**DOI:** 10.1371/journal.pone.0214557

**Published:** 2019-03-21

**Authors:** Davide Valeriani, Riccardo Poli

The affiliation for the second author is incorrect. Riccardo Poli is not affiliated with #2 but with #1: Brain Computer Interfaces and Neural Engineering Laboratory, School of Computer Science and Electronic Engineering, University of Essex, Colchester, United Kingdom.
